# Editorial: Genetic basis of tolerance induction defects underlying the development of autoimmune pathologies

**DOI:** 10.3389/fimmu.2022.1069232

**Published:** 2022-10-26

**Authors:** Jeremy J. Racine, Laurence Morel, Yi-Guang Chen, David V. Serreze

**Affiliations:** ^1^ The Jackson Laboratory, Bar Harbor, ME, United States; ^2^ Department of Microbiology, Immunology & Molecular Genetics, The University of Texas Health Sciences Center at San Antonio, San Antonio, TX, United States; ^3^ Department of Pediatrics, Medical College of Wisconsin, Milwaukee, WI, United States

**Keywords:** genetics of autoimmune diseases, NOD mouse, type 1 diabetes, Foxp3 regulatory T cells +, lupus (SLE), B cell tolerance, Mendelian randomization, polygenic autoimmune disease

In our initial call for review articles for the Research Topic “*Genetic Basis of Tolerance Induction Defects Underlying the Development of Autoimmune Pathologies*” we envisioned four areas of interest for this Research Topic: Area 1 - the genetic basis for tolerance induction defects underlying any single or multiple autoimmune disease states; Area 2 - the controversy concerning how well some animal models, in particular mice, inform the basis of various autoimmune pathologies in humans; Area 3 - how gene variants disrupt Treg/Breg development or activity resulting in autoimmune disease states; and Area 4 - genes x environment interactions contributing to various autoimmune states.

For anyone involved in autoimmunity research, it is not surprising to find the usual suspects in the three review articles that fall under Area 1 of this Topic: MHCs/HLAs, PTPN22, PTPN2, CTLA4, IFIH1 etc. The editors are grateful to the contributing authors for presenting these common players from differing perspectives using both overlapping and unique sources. Here, we would like to call out discussed allelic variants unique to each of the reviews. Kissler’s “*Genetic Modifiers of Thymic Selection and Central Tolerance in Type 1 Diabetes”* presents a section on *TAGAP* which may be of interest for those studying thymic migration of developing T-cells. Hocking and Buckner’s “*Genetic basis of defects in immune tolerance underlying the development of autoimmunity*” presents a section on *PADI2* and *PADI4* variants in rheumatoid arthritis (RA). *PADI2* and *PADI4* are enzymes responsible for the conversion of arginine to citrulline. Recent work has indicated that post translational modifications like citrullination can cause the creation of neoepitopes in type 1 diabetes [T1D) (reviewed in (1, 2)]. Finally, Gootjes et al.’s “*Functional Impact of Risk Gene Variants on the Autoimmune Responses in Type 1 Diabetes*” has a section dedicated to CD226. Researchers investigating the CD226 versus TIGIT axis may find this section of interest. Additionally, much of this section focuses on a particular CD226 associated SNP (rs763361) which has been implicated in multiple autoimmune disorders.

T1D researchers have long had to balance the power of the NOD mouse for dissecting the genetic and cellular contributions to T1D with the difficulty in clinical translation [reviewed 19 years apart in (3, 4)]. Three review articles submitted to this Research Topic loosely fall into the parameters of Area 2. Two of the most common complaints of the NOD mouse are: 1) The ease in preventing T1D development and 2) additional autoimmune manifestations beyond T1D. Aubin et al.’s “*The NOD Mouse Beyond Autoimmune Diabetes*” focuses on these other autoimmune manifestations, especially in the context of experimental manipulations that render the strain T1D-resistant. This review argues for the utility of the NOD as a model for understanding a diverse range of autoimmune disorders. Harley et al.’s “*Polygenic autoimmune disease risk alleles impacting B cell tolerance act in concert across shared molecular networks in mouse and in humans*” focuses on the use of risk-gene network analyses impacting B-cell tolerance utilizing T1D and SLE as models. This review showcases how network analyses can pinpoint where monogenic and polygenic versions of these diseases overlap, as well as the extent and areas human and murine disease networks may be similar or different. Additionally, the authors provide multiple possible explanations for the translation gap between mouse and humans with heavy focus on the limited environmental diversity presented to experimental mouse colonies versus the great variability in patients (Topic Area 4). Finally, Rojas et al.’s “*The long and winding road: From mouse linkage studies to a novel human therapeutic pathway in type 1 diabetes* ^1^” bridges Topic Areas 1, 2 and 3. Much of this review focuses on the still ongoing journey of identification of a T1D-susceptibility gene in NOD mice to developing and testing a putative future clinical therapeutic.

Two review articles in this Research Topic focus on the biology of Tregs with a special emphasis on their stability. Roach and Morel’s ^2^ article “*Genetic Variations Controlling Regulatory T Cell Development and Activity in Mouse Models of Lupus-Like Autoimmunity*” focuses on this topic in the context of SLE. One section that may be of particular interest delves into the genes that regulate Treg metabolism and how this may affect their functionality. Raugh et. al.’s “*Nature vs. nurture: FOXP3, genetics, and tissue environment shape Treg function*” provides a deep dive into the biology of Tregs covering diverse topics from their heterogeneity, to the genetic, epigenetic, and non-coding RNA control of the development and activity of these cells. Topic Area 4 is also touched upon, as the role of microenvironmental cues, such as microbiome, is briefly covered. Finally, this review discusses how these areas impact possible Treg-based therapy development.

Finally, while we set out to focus solely on review articles for this Topic, two primary research articles were submitted that the Editors felt sufficient to include within the scope of our original goals for this Topic. Di Lorenzo et al.’s “*Natural history of type 1 diabetes on an immunodysregulatory background with genetic alteration in B-cell activating factor receptor: A case report*” details the identification of a clinical case of T1D and common variable immunodeficiency in a patient with a low T1D-risk score putatively caused by the monoallelic H159Y mutation in *TNFRSF13C* (BAFFR). Zhong et al.’s “*Herpesvirus entry mediator on T cells as a protective factor for myasthenia gravis: A Mendelian randomization study*” utilized Mendelian Randomization to identify two SNPs (rs1886730 and rs2227313) in *TNFRSF14* associated with herpesvirus entry mediator (HVEM) expression on T-cells and protection from myasthenia gravis. This adds to the growing body of evidence on the role of HVEM - BTLA interactions in modulating autoimmune diseases, just recently reviewed in (5).

Together, the articles in this Research Topic provide an up-to-date overview on genetic contributions to immune tolerance pathways and autoimmunity.

## Author contributions

JJR, LM, Y-GC, DVS were all Topic Editors for this Research Topic and contributed to the preparation of this Editorial. All authors contributed to the article and approved the submitted version.

## Conflict of interest

The authors declare that the research was conducted in the absence of any commercial or financial relationships that could be construed as a potential conflict of interest.

## Publisher’s note

All claims expressed in this article are solely those of the authors and do not necessarily represent those of their affiliated organizations, or those of the publisher, the editors and the reviewers. Any product that may be evaluated in this article, or claim that may be made by its manufacturer, is not guaranteed or endorsed by the publisher.

## Footnotes

1 Yi-Guang Chen, a co-author on this editorial, is also a co-author on this cited review.

2 Laurence Morel is also a co-author on this editorial.
